# Denoising Method for NV-Center Fluorescence Signals Based on MPA-VMD Combined with Wavelet Thresholding

**DOI:** 10.3390/mi17030289

**Published:** 2026-02-26

**Authors:** Yanxin He, Xin Li, Zhonghao Li, Hao Guo, Huan Fei Wen, Jun Tang, Jun Liu

**Affiliations:** 1 State Key Laboratory of Extreme Environment Optoelectronic Dynamic Measurement Technology and Instrument, School of Instrument and Electronics, North University of China, Taiyuan 030051, China; heyanxin1010@163.com (Y.H.); lizh@nuc.edu.cn (Z.L.); guohao@nuc.edu.cn (H.G.); liuj@nuc.edu.cn (J.L.); 2 Science and Technology on Electronic Test and Measurement Laboratory, North University of China, Taiyuan 030051, China; 3 State Key Laboratory of Widegap Semiconductor Optoelectronic Materials and Technologies, North University of China, Taiyuan 030051, China; tangjun@nuc.edu.cn

**Keywords:** nitrogen-vacancy center, fluorescence signal denoising, variational mode decomposition, marine predators algorithm, wavelet thresholding, ODMR

## Abstract

To address complex noise in nitrogen-vacancy center fluorescence signal acquisition, a hybrid denoising framework combining marine predators algorithm-optimized variational mode decomposition (VMD) and wavelet thresholding is proposed. MPA adaptively selects VMD parameters, enhancing decomposition reliability. Wavelet thresholding then suppresses noise-dominant intrinsic mode functions while preserving signal components. Results show significant SNR improvement to 57.12 dB (14.6% higher than standalone VMD), RMSE reduction by 56.7%, and 7.9% SNR enhancement over wavelet thresholding alone, with the correlation coefficient reaching 0.97. More importantly, the proposed method substantially improves the accuracy of ODMR resonance parameter estimation. Compared to wavelet denoising, RMSE of the center frequency is reduced by 29.8% and RMSE of the FWHM is reduced by 44.5%; compared to VMD denoising, the FWHM RMSE is reduced by 20.7% while maintaining comparable center frequency accuracy. This approach validates the synergistic effect of VMD’s global decomposition and wavelet’s local denoising, offering an effective method for high-precision ODMR inversion with substantial application potential in quantum sensing and precision measurement.

## 1. Introduction

Nitrogen vacancy (NV) centers in diamond have emerged as a cornerstone platform for quantum sensing, leveraging their exceptional atomic-scale spatial resolution and room-temperature operational stability [1]. The pivotal technique of optically detected magnetic resonance (ODMR) enables quantum state interrogation by detecting fluorescence intensity variations in response to microwave frequency sweeps [2], with broad applications spanning condensed matter physics and biomedical imaging.

However, experimentally acquired fluorescence signals remain susceptible to various noise sources, including laser shot noise and microwave control crosstalk. These contaminants manifest as significant intensity fluctuations in recorded signals [3], leading to ODMR spectral linewidth broadening and resonance contrast reduction, which ultimately compromise the accuracy of magnetic field and temperature quantification. Therefore, developing effective denoising methods is not merely a signal-processing exercise, but a critical prerequisite for improving the sensitivity and precision of NV-based quantum sensors.

Conventional denoising approaches exhibit fundamental limitations when addressing the non-stationary characteristics of NV fluorescence signals, often resulting in incomplete noise suppression or distortion of critical signal features. Wavelet denoising [4] performance is critically dependent on the selection of mother wavelets and thresholding rules, rendering it inadequate for handling complex noise architectures. Empirical mode decomposition (EMD) [5] is susceptible to spurious components caused by mode mixing. Although variational mode decomposition (VMD) [6] mitigates certain EMD shortcomings through constrained optimization, its performance is highly sensitive to the predetermined number of modes (*K*) and the penalty factor (*α*). Suboptimal parameter selection may lead to under-decomposition with residual noise or over-decomposition [7] that compromises signal integrity.

To overcome these challenges, we propose a hybrid denoising framework that synergistically integrates the marine predators algorithm (MPA) [8] for optimizing VMD parameters with an enhanced wavelet thresholding technique. The MPA, a metaheuristic algorithm renowned for its efficient global search capability, is employed to autonomously determine the optimal VMD parameters (*K*, *α*), thereby enabling adaptive decomposition of the fluorescence signal into a series of intrinsic mode functions (IMFs) [9]. Subsequently, signal-dominant and noise-dominant IMFs are discriminated based on correlation coefficient criteria. An adaptive wavelet thresholding function [10] is then selectively applied to noise-dominant IMFs for effective noise suppression, while signal-dominant IMFs remain unaltered to preserve critical quantum information. Finally, the signal is reconstructed from the IMFs, achieving noise reduction through the synergy between global parameter optimization and local fine-grained denoising.

The innovation of this work lies in the intelligent integration of optimization algorithms with adaptive signal processing, harnessing their respective strengths: MPA provides global parameter optimization capability, VMD achieves physical signal decomposition, and wavelet thresholding enables localized denoising. Crucially, by improving the accuracy of ODMR resonance parameter estimation—particularly center frequency and linewidth—the proposed method directly enhances the core metrological performance of NV-based quantum sensors. This work thus bridges the gap between generic signal denoising techniques and task-specific quantum sensing requirements, demonstrating significant potential not only for NV center systems [11] but also for broader quantum sensing applications.

## 2. Theoretical Analysis

### 2.1. Variational Mode Decomposition (VMD)

VMD is a completely non-recursive adaptive complex signal-processing method [12]. By constructing and solving the constrained variational model, it aims to decompose the original signal *f*(*t*) into a series of intrinsic mode functions (IMFs) with independent center frequency and bandwidth. The objective is to minimize the sum of the estimated bandwidths of all modes, which is equivalently formulated as minimizing the squared L^2^-norm of the gradient of each demodulated mode.

The constrained variational model is given by:

(1) $$\left\{\begin{array}{l} \underset{\left\{u_{k}\},\{\omega_{k}\right\}}{\min}\{{\sum_{k}\left\|\partial_{t}[(\delta (t)+\frac{j}{\pi t})*u_{k}(t)]e^{-j\omega_{k}t}\right\|}_{2}^{2}\}\\ s.t.\sum_{k}u_{k}=f \end{array}\right.$$

where *u_k_* is the *k*-th IMF component, and *ω_k_* is its constant center frequency [13]. The term *e*^−*jω*^*_k_^t^* [14] shifts the frequency spectrum of each analytic mode to baseband. *δ*(*t*) is a Dirac function, ∂*_t_* is the partial derivative of time, and ${\left||.|\right|}_{2}^{2}$ denotes the square of the two-norm.

To solve this expression, the Alternating Direction Method of Multipliers (ADMM) [15] is employed. The modes {*u_k_*} and their center frequencies {*ω_k_*} are updated via the augmented Lagrangian function until the convergence criterion (e.g., *ϵ* ≤ 10^−6^) is satisfied.

Envelope spectral entropy is an index used to measure the complexity of signals. Its minimization indicates that the more regular the envelope distribution, the fewer noise components it contains. Therefore, this paper takes the minimum envelope spectrum entropy [16] as the objective of the fitness function, and its expression is:

(2) $$H_{en}=-\sum_{i=1}^{n}p_{i}\log p_{i}$$

where *p_i_* is the normalized probability density function and *n* is the number of envelope sampling points.

### 2.2. Marine Predators Algorithm (MPA) for VMD Parameter Optimization

To overcome the limitation of empirical parameter selection, we employ the marine predators algorithm (MPA) to autonomously search for the optimal parameter combination [*K*, *α*].

The MPA is a nature-inspired metaheuristic algorithm proposed by Faramarzi et al. [17], which simulates the foraging behavior of marine predators. The algorithm integrates Lévy flight and Brownian motion to achieve an effective balance between global exploration and local exploitation. Compared with other metaheuristic algorithms such as GWO and AO, the MPA offers faster convergence and higher optimization accuracy. For a detailed mathematical formulation of MPA, readers are referred to the original publication [17].

The fitness function for optimization is defined as the minimum envelope spectrum entropy of the decomposed IMFs, as given in Equation (2). The MPA iteratively updates the population to minimize this fitness value, thereby identifying the optimal VMD parameters.

### 2.3. Wavelet Threshold Denoising

**Figure 1:**
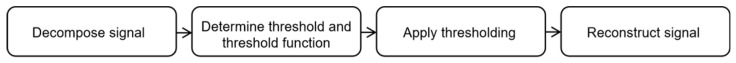
Workflow diagram of the wavelet threshold denoising algorithm.

Wavelet threshold denoising [18] is a signal-processing technology that uses the multi-resolution analysis characteristics of wavelet transform to realize the separation of noise and effective signal. Its core process is shown in Figure 1:

Firstly, the noisy signal is decomposed into approximate components (low frequency, retaining the main body of the signal) and detail components (high frequency, containing noise and detail) by wavelet basis function (db5). Subsequently, the ‘Heursure’ composite criterion is used to adaptively determine the threshold and the hard threshold function processing coefficient [19], aiming to suppress the noise while retaining the useful signal to the maximum extent. Finally, the results are subjected to inverse wavelet transform and reconstruction to obtain a noise reduction signal. This method can effectively distinguish signal and noise, and is suitable for denoising of non-stationary signals.

### 2.4. Denoising Method Based on MPA-Optimized VMD Combined with Wavelet Thresholding

#### 2.4.1. Complete Workflow of the Proposed Method

Based on the global decomposition of VMD and the local noise reduction characteristics of wavelet denoising, a hybrid framework integrating MPA-optimized VMD with wavelet thresholding is proposed to overcome limitations of conventional methods, including the endpoint effect in EMD, global–local imbalance in standalone VMD, and noise misjudgment in wavelet thresholding (WT): MPA dynamically optimizes VMD parameters to avoid decomposition distortion. On this basis, the wavelet threshold only suppresses the noise IMF and retains the high-frequency characteristics of the signal. Compared with a single method, the hybrid strategy aims to improve the decomposition stability and noise suppression accuracy through global–local coordination, and provides a systematic denoising scheme for complex noisy signal denoising.

**Figure 2:**
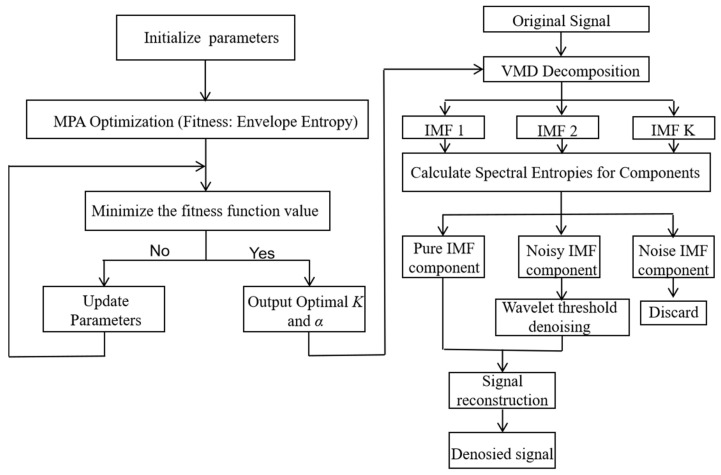
Flowchart of the denoising method based on MPA-optimized VMD combined with wavelet thresholding.

The method adopted in this paper is based on MPA-VMD and combined with wavelet threshold for noise reduction. The flowchart is shown in Figure 2:

(a) Initialize MPA parameters with population size = 50 and maximum iterations = 30, and define search ranges for VMD parameters as number of modes *K* ∈ [3, 11] and penalty factor *α* ∈ [10, 3000].
(b) Use MPA’s own advantages [20] to optimize the VMD parameters *K* and *α*, and the minimum envelope spectrum entropy value is used as the loop termination condition of the fitness function, and the optimal parameter combination is obtained by iterative coverage.
(c) Under this optimal combination, the VMD decomposition signal is started to obtain multiple IMF components.
(d) Calculate the power spectral entropy [21], sample entropy [22], variance contribution rate [23], and other indicators of each IMF component, so as to screen out the pure IMF component, the IMF component containing noise, and the noise IMF component.
(e) Wavelet threshold denoising is performed on the IMF component containing noise, and then the signal is reconstructed with the pure signal IMF component to achieve noise reduction; the noise IMF component is useless and directly discarded.
(f) Reconstruct the denoised signal by summing all processed IMFs.

#### 2.4.2. Quantitative Evaluation Metrics

To objectively assess the denoising performance, three complementary metrics are adopted:

Signal-to-Noise Ratio (SNR):

(3) $$SNR=10\log_{10}(\frac{\sum_{i=1}^{n}x^{2}(i)}{\sum_{i=1}^{n}{\left[x(i)-y(i)\right]}^{2}})$$

Cross-Correlation Coefficient (CC):

(4) $$CC=\frac{\sum_{i=1}^{n}\left(x(i)-\bar{x})(y(i)-\bar{y}\right)}{\sqrt{\sum_{i=1}^{n}{\left(x(i)-\bar{x}\right)}^{2}}\sqrt{\sum_{i=1}^{n}{\left(y(i)-\bar{y}\right)}^{2}}}$$

Root Mean Square Error (RMSE):

(5) $$RMSE=\sqrt{\frac{\sum_{i=1}^{n}{\left[x(i)-y(i)\right]}^{2}}{n}}$$

where *n* represents the total number, *x*(*i*) represents the source signal, and *y*(*i*) represents the denoised signal. The results show that the larger the SNR, the closer the CC is to 1, the smaller the RMSE, the better the denoising performance, and the stronger the signal consistency.

## 3. NV Fluorescence Signal Acquisition and Denoising Analysis

### 3.1. Experimental Setup and Dataset Description

#### 3.1.1. ODMR Signal Acquisition

The nitrogen-vacancy color center is a point defect with excellent spin characteristics in diamond. Its ground state is a spin triplet state. Zeeman splitting occurs under the action of an external magnetic field, resulting in a characteristic resonance peak in the optically detected magnetic resonance spectrum. When the applied microwave frequency resonates with the NV color center ground state energy level transition (*m_s_* = 0 ↔ *m_s_* = ±1), the electron spin population flips, and some electrons enter the *m_s_* = ±1 state and relax through the non-radiative pathway, resulting in a decrease in the overall fluorescence intensity. Optical detection magnetic resonance (ODMR) is the key technology to read the spin state of the NV color center. The resonance spectrum is obtained by scanning the microwave frequency [24], and then the physical quantities such as magnetic field and temperature are inverted.

**Figure 3:**
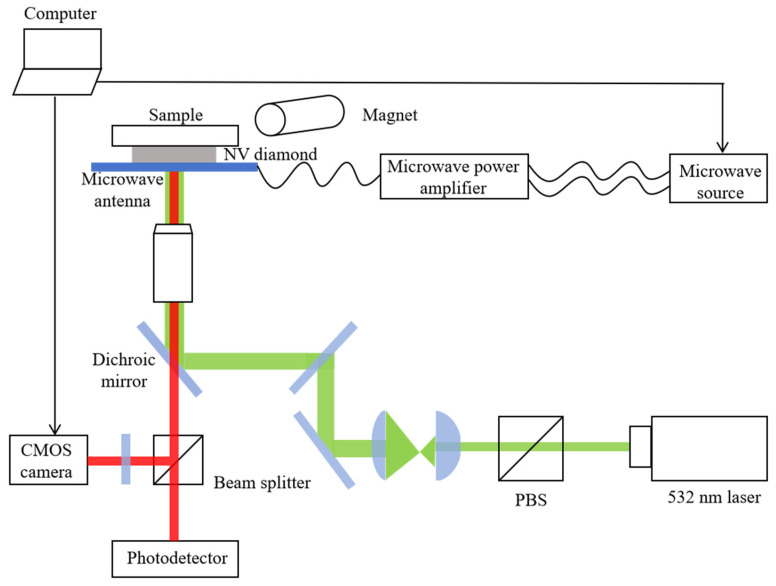
Overall block diagram of the NV fluorescence imaging system.

The specific process of collecting the ODMR signal is shown in Figure 3:

(a) Frequency sweep and imaging: The microwave frequency is scanned over a 2.5–3.2 GHz range using a microwave generator (Siglent, SDG1062X, Shenzhen, China). The CMOS camera (Thorlabs, CS505MU, Newton, NJ, USA) collects a fluorescence intensity image synchronously for each microwave frequency point. The cycle repeats until the full frequency sweep is complete.
(b) Data cube construction: The images from all frequency points are stacked into a 3D data cube *I*(*x*, *y*, *ν*), where *x*, *y* are spatial coordinates and *ν* is frequency coordinates.
(c) The extraction of ODMR spectra: The fluorescence intensity of any single pixel (*x*_0_, *y*_0_) at all frequencies is extracted from the cube, and an ODMR spectrum of the point is obtained, that is, the curve *I*(*ν*) of the fluorescence intensity changing with the microwave frequency.

Ideally, the optically detected magnetic resonance (ODMR) signal of the nitrogen vacancy (NV) color center can be modeled as a superposition of multi-peak Gaussian functions [25]:

(6) $$I(\nu )=I_{0}\left[1-\sum_{i=1}^{n}C_{i}\cdot \exp(-\frac{{\left(\nu -\nu_{i}\right)}^{2}}{{2\tau_{i}}^{2}})\right]$$

where *I*_0_ is the fluorescence intensity under non-resonant conditions, *C_i_* is the contrast of the i-th resonance peak, ν_i_ is the center frequency of the i-th resonance peak, and *τ_i_* is the standard deviation of the i-th Gaussian component. The full width at half maximum (FWHM) of the i-th resonance peak is given by (FWHM*_i_* ≈ 2.3548 · *τ_i_*).

**Figure 4:**
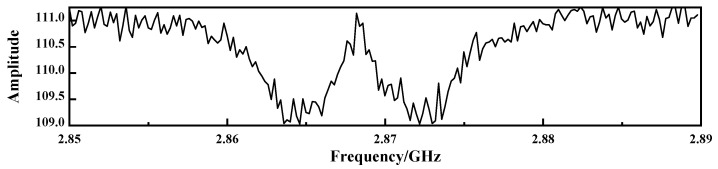
Raw ODMR signal extracted from a single pixel with frequency range 2.85–2.89 GHz.

However, as shown in Figure 4, experimentally measured ODMR signals inevitably deviate from the ideal model due to the influence of noise, showing significant amplitude jitter, frequency drift, and baseline fluctuation, which leads to resonance broadening and contrast reduction.

#### 3.1.2. Dataset Description

To comprehensively evaluate the generalization capability of the proposed method under different signal conditions, a dataset consisting of 55 ODMR spectra [26] acquired under varying experimental conditions was constructed. Each spectrum contains 41 frequency points covering the range of 2.85 GHz to 2.89 GHz.

Due to differences in measurement conditions (e.g., temperature) that affect the zero-field splitting of NV centers, these 55 spectra exhibit natural variation in their resonance characteristics. Such variation provides a representative testbed for assessing the robustness of the proposed denoising approach.

From this set of 55 spectra, 50 signals were randomly selected to form a validation dataset. All signals were preprocessed with baseline correction and normalization prior to denoising and analysis.

### 3.2. Analysis of the Optimization Process

#### 3.2.1. Selection and Demonstration of Optimization Algorithm

**Figure 5:**
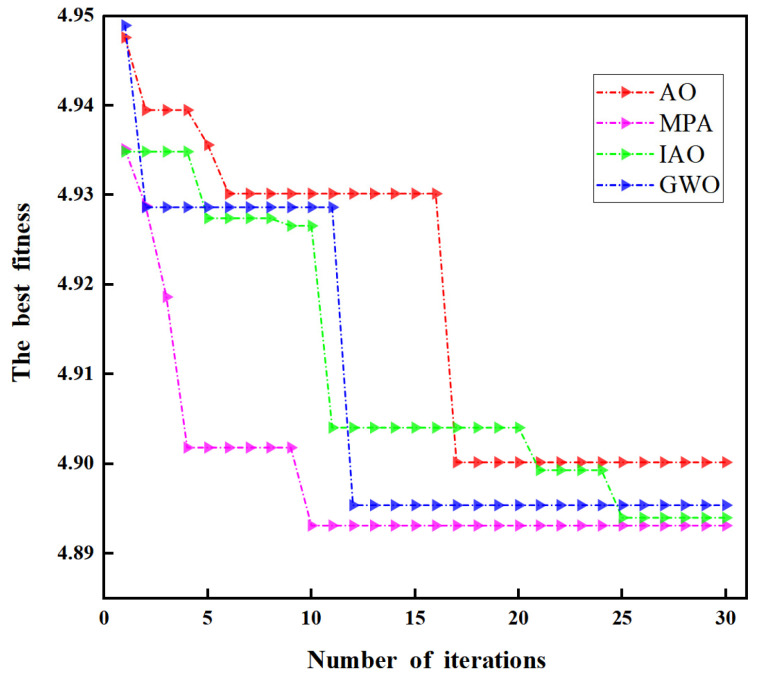
Iterative convergence curves of different optimization algorithms (GWO, AO, IAO, and MPA).

Firstly, we compared the MPA algorithm with the current mainstream meta-heuristic optimizers (such as Grey Wolf Optimizer (GWO) [27] and the Aquila Optimizer (AO) [28]). After repeated verification, the fitness function value obtained by the MPA algorithm was the lowest, so we decided to adopt the MPA algorithm, and the comparison results are shown in Figure 5.

As shown in the figure above, the GWO algorithm reaches a fitness value of approximately 4.896 around the 12th iteration. The AO algorithm converges at the 17th iteration with a fitness value of about 4.899. The improved Aquila Optimizer (IAO) converges only at the 25th iteration with a fitness value of approximately 4.894. In contrast, the marine predators algorithm (MPA) converges by the 10th iteration, achieving the lowest fitness value of about 4.893. Therefore, the MPA algorithm obtained the smallest fitness value, with the corresponding optimal parameter combination for *K* and *α* being 4 and 1500, respectively.

#### 3.2.2. Modal Decomposition and Screening

**Figure 6:**
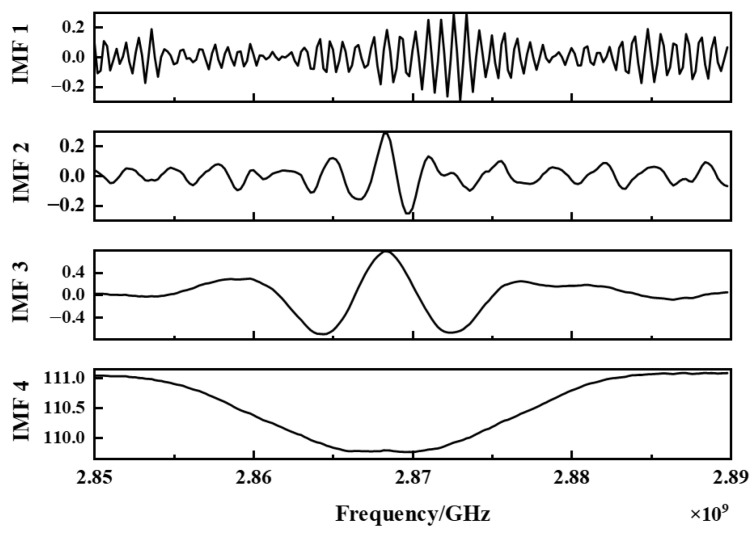
IMF components decomposed by MPA-VMD.

Using the optimized parameter combination [K, α] obtained in the first stage, the original NV fluorescence spectrum is decomposed by VMD, and five IMF components with limited bandwidth are obtained in the frequency domain. Figure 6 shows the power spectral density [29] (PSD) curve of each IMF component.

Noise-dominant mode (such as IMF1, IMF2): The PSD curve of this mode shows a broad and flat distribution, and the energy is not concentrated at any specific frequency [30].

The dominant mode of signal (such as IMF4): Unlike IMF1, its PSD curve shows a sharp and steep spectral peak at the zero-field splitting characteristic frequency (~2.87 GHz) of the NV color center, indicating that the mode successfully captures the core physical signal of the target.

Mixed mode (such as IMF3): its PSD shape is between noise and signal, and may contain sidebands or residual noise. To avoid loss of critical information, these components are retained.

To validate the above hypothesis, we established an objective criterion for IMF selection based on quantitative metrics. The power spectral entropy [21], sample entropy [22], and variance contribution rate [23] of each IMF component are listed in Table 1.

As shown in Table 1: IMF1 and IMF2: These two components have higher power spectral entropy (>3) and sample entropy (>0.7), indicating that their frequency energy distribution is the most disordered; at the same time, their variance contribution rate is the lowest (<0.1), indicating that their spectrum shape is the most different from the overall signal [31]. These indicators together determine that they are both noise-dominated modes. IMF3: The power spectral entropy (2.2736) of this component is between IMF1, IMF2, and IMF4, and the sample entropy (0.24936) is significantly lower than the corresponding sample entropy of the first two components [32]. Therefore, IMF3 can be judged as a residual mode. IMF4: The power spectral entropy (0.00019019) and sample entropy (0.10613) of this component are much lower than the other components, indicating that its energy is highly concentrated in a single frequency; at the same time, it has the highest variance contribution rate (0.6872). These indicators strongly prove that IMF 4 is the signal-dominant mode.

#### 3.2.3. Wavelet Threshold Processing and Signal Reconstruction

After obtaining the IMF obtained by MPA-VMD decomposition, we apply wavelet threshold processing to the IMF (IMF1, IMF2) determined to be noise-dominated by step (2), and completely retain the signal-dominated IMF. Next, the processed noise IMF is reconstructed by the inverse wavelet transform [33], and it is superimposed with the unprocessed signal dominant IMF to obtain a complete denoising signal.

**Figure 7:**
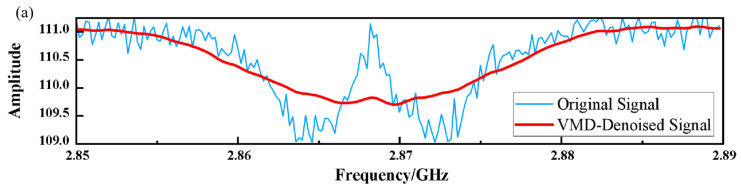

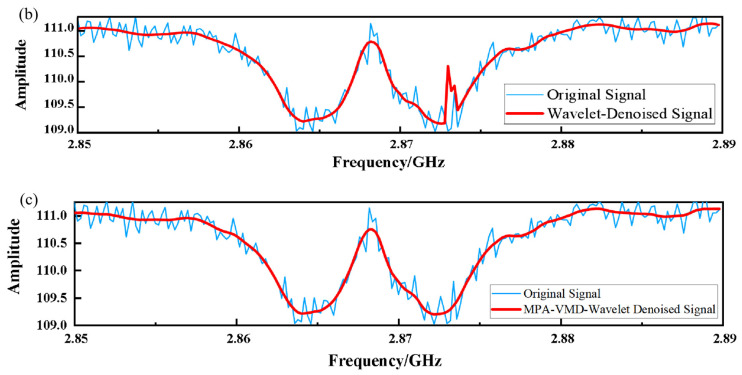
Comparison of denoising performance: (**a**) VMD denoising. (**b**) Wavelet threshold denoising. (**c**) Proposed MPA-VMD–wavelet method.

This ensures that the coherent physical signals concentrated in the narrow frequency band are completely free from pollution in the denoising process, while the incoherent broadband noise is effectively attenuated. After the above steps, the final comparison results before and after noise reduction are as follows:

Figure 7 compares the denoising performance of three methods. As shown in Figure 7a, VMD denoising removes excessive signal components, causing noticeable deviation from the original signal. Wavelet denoising (Figure 7b) reduces noise but retains an abnormal fluctuation around 2.873 GHz. In contrast, the proposed MPA-VMD–wavelet method (Figure 7c) achieves the best noise suppression while preserving the signal shape.

### 3.3. Denoising Performance Evaluation

#### 3.3.1. SNR, CC, and RMSE Comparison

Table 2 presents the quantitative performance comparison of three denoising methods applied to the ODMR signal shown in Figure 4.

From the denoising results in Table 2, the proposed method achieves the highest SNR of 57.12 dB, which is 14.6% higher than VMD denoising (49.85 dB) and 7.9% higher than wavelet thresholding (52.92 dB). Moreover, it yields the lowest RMSE of 0.1539, representing reductions of 56.7% and 29.3% compared to VMD (0.3556) and wavelet denoising (0.2176), respectively. These results demonstrate that the proposed method outperforms both standalone techniques in denoising performance.

#### 3.3.2. ODMR Parameter Estimation Accuracy

To validate the suitability of the proposed method for the core task of ODMR signal processing, we evaluated the accuracy of resonance parameter extraction after denoising. Using the Gaussian model fitting (Equation (6)), we calculated the center frequency and FWHM for a representative ODMR signal after applying different denoising methods [26].

The fitting results obtained from the raw signal were used as the reference baseline, and the deviations of each denoising method from this baseline were calculated. This approach allows quantitative comparison of the relative improvements achieved by different denoising techniques.

The results from Table 3 reveal that for center-frequency estimation, our method yields the lowest error (33.77 kHz), outperforming VMD-denoising (34.25 kHz) and substantially surpassing wavelet-denoising (48.07 kHz), with a reduction of 29.8%. For FWHM estimation, our method also achieves the best result (193.9 Hz), representing reductions of 20.7% and 44.5% compared to VMD- and wavelet-denoising, respectively.

These findings confirm that the proposed approach not only effectively suppresses noise but also better preserves the resonance line shape, delivering optimal accuracy for extracting both key parameters. It is therefore well-suited for high-precision NV-based quantum sensing that demands stringent parameter accuracy.

### 3.4. Statistical Validation and Generalization Performance

To assess the statistical significance and generalization capability of the proposed method, we conducted experiments on the full dataset of 50 experimental ODMR signals described in Section 3.1.2. Each signal was independently processed using four different methods, and the center frequency and FWHM were extracted via Gaussian model fitting in Equation (6).

In the same way, The fitting results from the raw signal were used as the reference baseline for each signal. Table 4 summarizes the statistical results of the 50 independent experiments.

As shown in Table 4, the center frequency obtained by the proposed method is 2.8732 GHz, which is closest to the fitting result of the raw signal (2.8738 GHz), with a deviation of only 0.6 MHz. In comparison, the deviation of the VMD-denoised result is 8.6 MHz, and that of the wavelet-denoised result is as high as 28.7 MHz. Meanwhile, the proposed method yields the smallest standard deviation (10.8 MHz), which is 25.5% lower than that of the raw signal (14.5 MHz) and notably lower than those of VMD-based (11.9 MHz) and wavelet-based (14.7 MHz) denoising, indicating that the proposed method provides more consistent results across different signals.

These results demonstrate that the proposed method effectively preserves the characteristics of the original signal while exhibiting excellent stability, thereby offering a suitable solution for high-precision NV-based quantum sensing applications.

While lock-in amplification is a powerful hardware-based denoising technique widely used in NV center measurements, our proposed method focuses on software-based post-processing that can be applied without modifying the experimental setup. These two approaches are complementary and could potentially be combined in future work to further enhance signal quality.

Lock-in amplification is a robust hardware-based denoising technique widely employed in NV-center measurements, whereas the method proposed in this work focuses on software-based post-processing that can be applied without altering the experimental setup. These two approaches are complementary and could be integrated in future studies to further improve signal quality.

## 4. Conclusions

A hybrid MPA-VMD–wavelet method is developed for denoising NV-center fluorescence signals. MPA optimizes VMD parameters (K = 4, α = 1500), enhancing mode decomposition. Selective wavelet thresholding is then applied to noise-dominant IMFs, effectively suppressing noise while preserving signal structure.

For a single ODMR signal, the SNR reaches 57.12 dB—14.6% higher than VMD alone and 7.9% higher than wavelet denoising alone. More critically, parameter estimation accuracy is substantially improved: compared to wavelet denoising, center-frequency RMSE decreases by 29.8% and FWHM RMSE by 44.5%. While center-frequency accuracy remains comparable to standalone VMD, FWHM RMSE is reduced by 20.7%.

Statistical validation across 50 signals under varied conditions shows that the proposed method yields an average center frequency closest to the raw signal (2.8732 ± 0.0108 GHz, deviation = 0.6 MHz) with the smallest standard deviation.

In summary, the method successfully integrates the global decomposition of VMD with the localized denoising of wavelet thresholding, enabling precise estimation of center frequency and FWHM, and offers a reliable signal-processing solution for high-accuracy quantum sensing.

## Figures and Tables

**Table 1:** Index table of IMF components.

	Power Spectral Entropy	Sample Entropy	Variance Contribution Rate
IMF 1	3.6587	1.0791	0.01594
IMF 2	3.8046	1.1704	0.025665
IMF 3	2.2736	0.24936	0.27119
IMF 4	0.00019019	0.10613	0.6872

**Table 2:** Performance comparison of different denoising methods.

Method	SNR (dB)	CC	RMSE
VMD denoising	49.85	0.8506	0.3556
Wavelet thresholding denoising	52.92	0.9516	0.2176
The proposed method	57.12	0.9730	0.1539

**Table 3:** ODMR resonance parameter estimation errors of different methods.

Method	Center Frequency RMSE (Hz)	FWHM RMSE (Hz)
VMD denoising + fitting	34,245	244.57
Wavelet denoising + fitting	48,074	349.23
Proposed method + fitting	33,770	193.93

**Table 4:** Statistical comparison of center frequency deviations for 50 ODMR signals (mean ± std).

Method	Center Frequency Deviation (GHz)
Raw signal fitting	2.8738 ± 0.0145
VMD denoising + fitting	2.8824 ± 0.0119
Wavelet denoising + fitting	2.9025 ± 0.0147
Proposed method + fitting	2.8732 ± 0.0108

## Data Availability

The raw data supporting the conclusions of this article will be made available by the authors on request.
